# Investigating the benthic megafauna in the eastern Clarion Clipperton Fracture Zone (north-east Pacific) based on distribution models predicted with random forest

**DOI:** 10.1038/s41598-022-12323-0

**Published:** 2022-05-17

**Authors:** Katja Uhlenkott, Erik Simon-Lledó, Annemiek Vink, Pedro Martínez Arbizu

**Affiliations:** 1 German Centre for Marine Biodiversity Research (DZMB), Senckenberg am Meer, Südstrand 44, 26382 Wilhelmshaven, Germany; 2 Marine Biodiversity Research, Institute for Biology and Environmental Sciences, Carl Von Ossietzky University Oldenburg, 26111 Oldenburg, Germany; 3 National Oceanography Centre, Empress Dock, Southampton, SO14 3ZH UK; 4 Federal Institute for Geosciences and Natural Resources (BGR), Stilleweg 2, 30655 Hannover, Germany

**Keywords:** Computational biology and bioinformatics, Ecology, Zoology, Ecology, Environmental sciences, Ocean sciences

## Abstract

The eastern Clarion Clipperton Fracture Zone (CCZ) is a heterogeneous abyssal environment harbouring relatively low abundances of highly diverse megafauna communities. Potential future mining of polymetallic nodules threatens these benthic communities and calls for detailed spatial investigation of megafauna. Based on the predicted probability of occurrence of 68 megafauna morphotypes, a seabed area extending over 62,000 km^2^ was divided into three assemblages covering an eastern plain area, a deeper western plain area and an area covering both seamount and abyssal hill sites. Richness, estimated as the sum of morphotypes with a predicted probability of occurrence larger than 0.5, amounts to 15.4 of 68 morphotypes. Highest richness was predicted at seamount sites, and lowest richness in the western part of the study area. Combining the predicted probability of megafauna occurrences with bathymetric variables, two seamount habitats and two plain habitats could be defined. One of these megafauna plain habitats corresponds with contiguous nodule fields of high abundance that may be targeted for future mining, showing that prospective nodule fields have a clearly differentiated megafauna assemblage. Monitoring and management schemes, including the delineation of preservation and protection areas within contract areas, need to incorporate this geological and biological heterogeneity.

## Introduction

The Clarion Clipperton Fracture Zone (CCZ, NE Pacific) is a heterogeneous abyssal plain area characterized by differences in depth, productivity, and topographic complexity^1^. The relatively high heterogeneity of the CCZ seabed is thought to promote the development of highly diverse benthic communities^2^. Distributions of benthic megafauna (animals detectable in seabed images^3^, typically > 1 cm) are known to vary across different habitats in the CCZ, both locally and regionally^4^. Abundance and composition of megafauna assemblages are thought to be partly driven by changes in the terrain, as substantially different communities usually occur in trough, plain, and hill areas^4,5^. Furthermore, the CCZ is known for its high abundances of polymetallic nodules, ore concretions composed of relatively high quantities of critical metals, on the seafloor^6,7^. Nodule fields extend over vast areas, which has attracted the interest of the newly developing deep-sea mining industry^7^. The association between fauna and the hard substratum provided by the nodules in the abyssal plains appears to play a central role in the structuring of megabenthic communities in the CCZ^4,8^. Nevertheless, the International Seabed Authority (ISA) had granted 16 exploration licenses for polymetallic nodules in the CCZ by early 2021^9^, despite the fact that the ecological and taxonomical understanding of these ecosystems is limited and predictions that severe and long-lasting effects of mining activities on benthic communities will occur^10,11^.

The seabed area targeted in this study is the German contract area for exploration of polymetallic nodules in the eastern CCZ, which is characterized by extensive abyssal fields with nodules and smaller fields without nodule coverage as well as numerous seamounts and abyssal hills^12^. Variations in abundance and composition of megafauna across local nodule availability gradients (e.g. within tens of meters) were found to be of comparable magnitude to those observed at more regional scales (e.g. within hundreds of km)^4^. Especially nodule-free areas harbour distinctly lower abundances of both mobile and sessile megafauna compared to areas with nodule coverage^8,13^ as a large number of megabenthic taxa usually only occur on the nodules themselves^5,14^. For instance, almost 70% of the suspension feeders observed in the APEI-6, a marine protected area north of our study area, are commonly found attached to hard substratum^5^. However, hard substratum is not only provided by polymetallic nodules; rock outcrops typically found in conjunction with seamounts and abyssal hills are another source of hard substratum in the CCZ^12^. Due to their generally high biodiversity^15^, a strong focus was placed on the presence of seamounts during the proposition of protected areas in the context of nodule mining^1^, e.g. to act as potential reservoirs for key species^16^. However, community composition has been shown to differ substantially when comparing seamount areas to neighbouring nodule fields in the CCZ^17^.

On a regional scale, a system of areas of particular environmental interest (APEIs) has been established by the ISA as a scheme of protected areas, i.e. no-mining zones, surrounding the contract areas for the exploration of polymetallic nodules in the centre of the CCZ^18^. On the smaller scale of a contract area, the ISA regulations prescribe that contractors delineate so-called preservation reference zones together with potential impact areas^19,20^. These preservation reference zones, where no mining shall occur, should function as control sites to accurately monitor the effects of mining disturbance in nearby impacted sites and should hence be comparable in size and habitat composition (including the mineral resource quality) to the impacted zones^21^. In addition to preservation reference zones, protected areas with similar conditions to potential mining areas within contract areas may also be highly important for conservation purposes^22^. Consequently, there is an urgent need for methodology and regulation that allow a robust delineation of preservation and impact reference zones and potential biodiversity hotspots, and thus for tools that effectively define different habitats within and beyond contract areas.

An approach based on the clustering of predicted faunal distributions and environmental variables has been suggested as a useful tool to define different habitats within an exploration contract area^22^. Species distribution models are used to investigate how different environmental drivers, or the combination of these, structure benthic communities spatially across at the CCZ. The algorithm random forest^23^, which is based on decision trees, has shown to be robust and effective for the investigation of spatial patterns in the distribution of deep-sea meio- and macrofauna^12,22,24,25^ as well as the distribution of polymetallic nodule abundances^26^. Empirically, this method often outperforms other common methods, e.g. Generalized Linear Models, and is especially useful for prediction^27^.

A previous study conducted in the German contract area assessed the use of meiofauna distribution models for the delineation of habitats^22^. As commonly observed in deep-sea environments, meiofaunal organisms occur in high abundances of several hundred individuals per square decimetre in the surface sediment of the CCZ^25,28–31^. In contrast, megafauna occurs in low abundances of often less than one individual per square meter in the CCZ^4,5,14^. However, their distribution patterns can be monitored using seabed image surveys across comparably much larger areas^4,5^ and at a relatively low cost^3^. Due to the high habitat heterogeneity and despite the low abundances, there are large differences in the lifestyle of CCZ megafaunal organisms. The high diversity and wide range of lifestyles of CCZ megafauna appears to promote a wide variety of distribution patterns in these communities (e.g.^4^), making this a highly suitable size class for distribution modelling.

In this study, we apply the random forest algorithm to predict spatially distributions of megafauna morphotypes across different geomorphological units found within the eastern German exploration area in the CCZ. Based on these predictions, we use k-means clustering to detect areas with similar megafauna communities as well as environmental conditions, which can potentially serve to delineate preservation and impact zones within a contract area, along with the detection of areas that may potentially need additional, specific protection (e.g. biodiversity hotspots). We discuss our results in the light of previous findings derived from the application of the same approach on meiofauna distributions^22,32^.

## Results

### Megafauna assemblages

In the study area, which corresponds to the German contract area for the exploration of polymetallic nodules, a total of 11,672 metazoan invertebrate megafauna specimens were detected from seabed images (encompassing a seafloor area of 18,439 m^2^), resulting in an average density of 0.6 individuals per square meter. Of these, 6247 specimens could be assigned to 208 different morphotypes (including 68 singletons) belonging to 25 higher taxa (e.g. Actiniaria, Hexactinellida, Ophiuroidea). Excluding all morphotypes occurring at less than 10 images (i.e. 140 taxa), a total of 68 morphotypes (encompassing 5838 specimens) were used as response variables for random forest classification (s. Supplementary Table S1).

**Figure 1:**
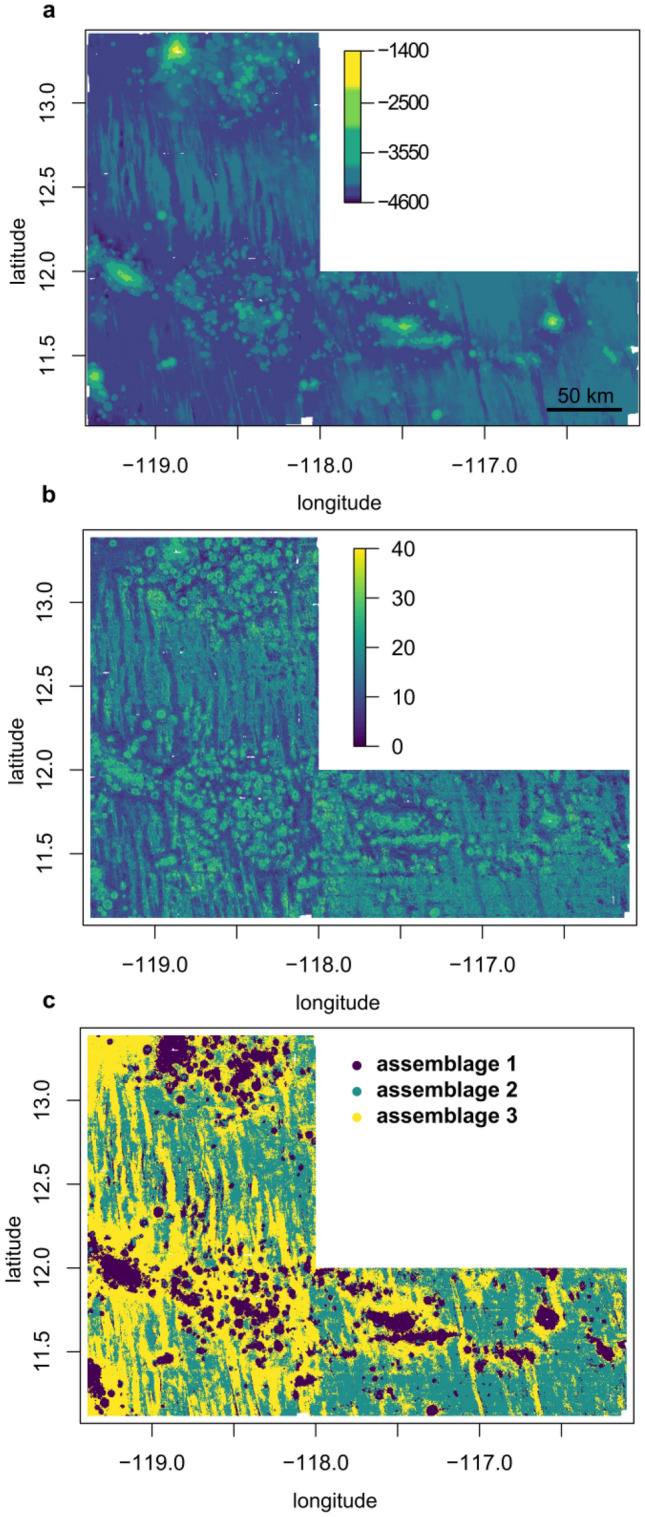
Map of the study area showing water depth (**a**), morphotype richness as the summarized number of morphotypes with a predicted probability of more than 0.5 at each position (**b**), and the megafauna assemblages computed with k-means clustering based on the predicted probability of occurrence of the megafauna morphotypes (**c**).

Based on the random forest models, the probability of occurrence of each morphotype was predicted across the study area individually. Combining the number of morphotypes with a predicted probability of occurrence of > 0.5 per cell as an approximation of potential morphotype richness, the mean predicted richness across the study area was 15.4 ± 6.1 (out of the total 68) morphotypes per cell. Regarding the predictions spatially, richness was predicted to be especially high at seamount sites and low in abyssal plain areas in close proximity to seamount sites (Fig. 1a,b). Generally, predicted richness in abyssal plain areas was higher in the eastern part of the study area compared to the west (Fig. 1b).

Clustering the probabilities of megafauna occurrences with k-means clustering, the community was divided into 3 distinct assemblages (Fig. 1c) based on the Calinski criterion^33^. Using PERMANOVA to pairwise compare the different clusters, the predicted probability of megafauna occurrences differed significantly between all three assemblages (adjusted p-value: 0.003) (s. Supplementary Table S2). While assemblage 1 corresponds to seamount sites and abyssal hills, assemblage 3 is mainly located in the surroundings of the seamounts and in the deeper areas in the west of the study area (Fig. 1a,c). Assemblage 2 is most prevalent in the east of the study area as well as between the two seamount chains in the north-west (Fig. 1c).

Assemblage 1, including seamount sites, comprises the lowest water depth, with a minimum of 1468 m and a maximum of 4454 m, and a mean depth of 3978 m (Table 1). This assemblage includes the locations of 488 images used for the investigation of megafauna composition (Table 1). Assemblage 3, usually surrounding assemblage 1, comprises the deepest depth range, between 3242 and 4572 m, with a mean depth of 4321 m (Table 1). Assemblage 2 encompasses a mean water depth of 4229 m and the lowest standard deviation of water depth (Table 1), with a maximum water depth of 4480 m and minimum of 2536 m. Assemblage 2 and 3 contain the locations of a similar amount of images (2127 and 2132 images, respectively) (Table 1).

**Table 1:** Comparison of the different megafauna assemblages computed with k-means clustering (Fig. 1c) based on the predicted probability of occurrences of megafauna morphotypes across the whole study area; total richness refers to the original dataset including all morphotypes with no regard if they could be used in the modelling approach or if the numbers of occurrences were too low.

	Assemblage 1	Assemblage 2	Assemblage 3
Total richness	112	169	140
Data points	488	2127	2132
Water depth	3978 ± 307 m	4229 ± 60 m	4321 ± 77 m
n images	488	2127	2132
n specimens	1222	2901	2124

**Figure 2:**
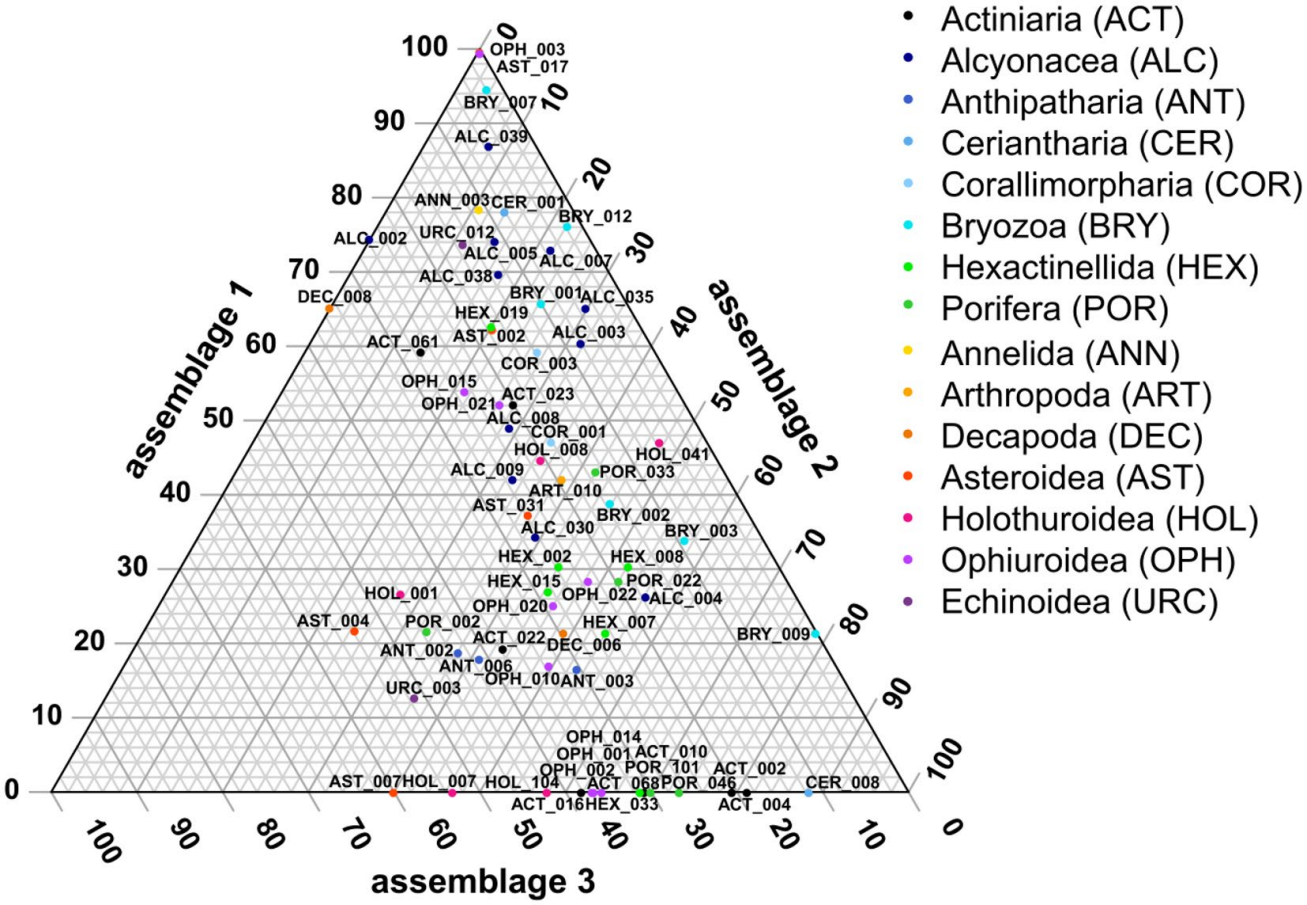
Ternary plot of the percentage occurrences per assemblage of the 68 megafauna morphotypes occurring in more than 10 images (Fig. 1). Only morphotype codes are given, for the complete names see Supplementary Table S1.

**Figure 3:**
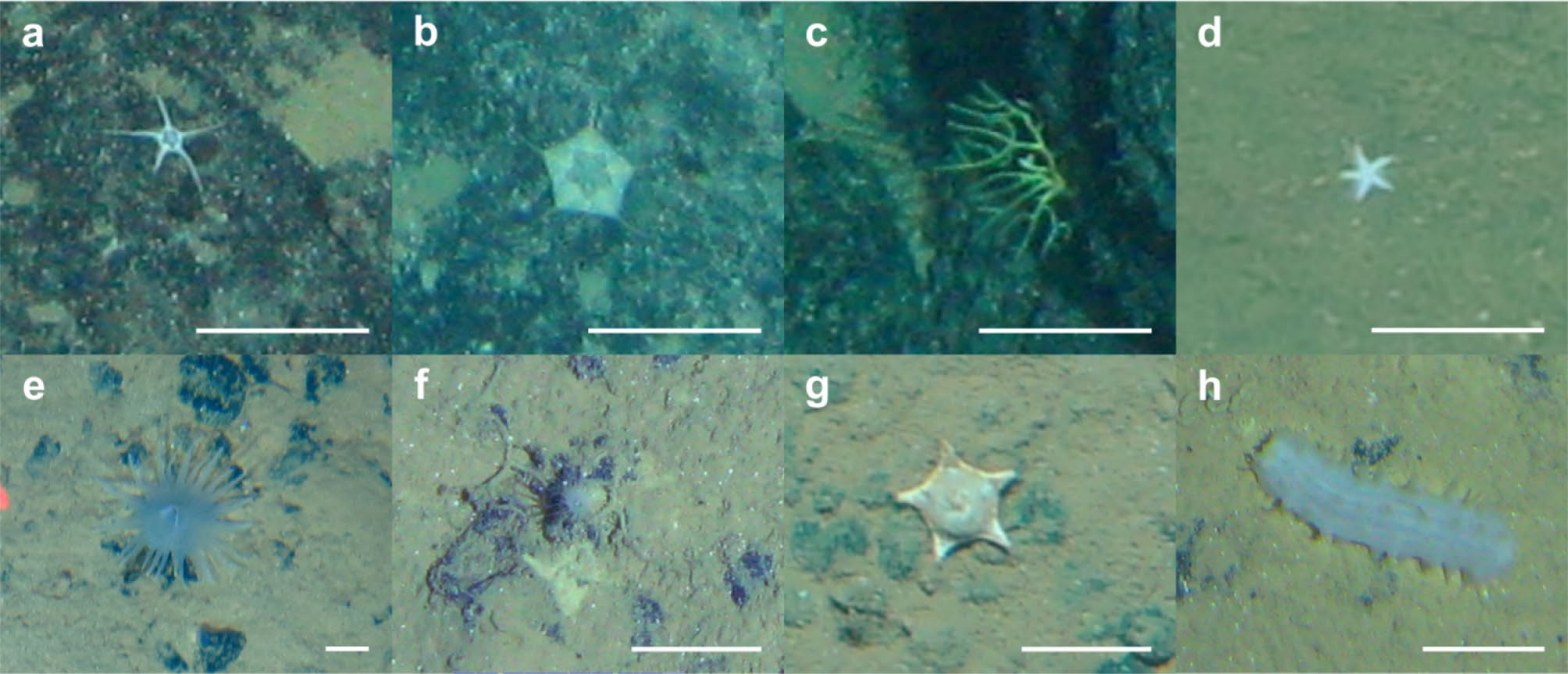
Images of selected morphotypes most closely associated with an assemblage: Ophiopyrgidae mtp-OPH_003 (**a**), *Hymenaster* sp. indet. mtp-AST_017 (**b**), Bryozoa mtp-BRY_007 (**c**), Paxillosida mtp-AST_004 (**d**), Actiniaria mtp-ACT_004 (**e**), Ceriantharia mtp-CER_008 (**f**), *Hyphalaster* sp. indet. mtp-AST_007 (**g**), *Synallactes* sp. indet. mtp-HOL_007 (**h**); scale bars represent 5 cm.

Total morphotype richness, i.e. the number of morphotypes observed within the locations mapped as a given assemblage type, was highest in assemblage 2 with 169 morphotypes, followed by assemblage 3 with 140 morphotypes, while assemblage 1 harboured 112 morphotypes. Investigating the distribution of the 68 most frequent morphotypes across the three assemblages, the morphotypes contributing most to differences between assemblages were Ophiopyrgidae mtp-OPH_003 (Fig. 3a) and *Hymenaster* sp. indet. mtp-AST_017 (Fig. 3b), which only occur in assemblage 1 (Fig. 2). Another morphotype that is closely assigned to assemblage 1 is Bryozoa mtp-BRY_007 (Figs. 2, 3c). A large number is also absent from assemblage 1, with Ceriantharia mtp-CER_008 (Fig. 3f) and Actiniaria mtp-ACT_004 (Fig. 3e) being most closely assigned specifically to assemblage 2 (Fig. 2). There were no morphotypes close to being specifically observed within assemblage 3, the most closely assigned morphotypes being the asteroids *Hyphalaster* sp. indet. mtp-AST_007 (Fig. 3g) and Paxillosida mtp-AST_004 (Fig. 3d) (Fig. 2) as well as the holothurian *Synallactes* sp. indet. mtp-HOL_007 (Fig. 3h).

### The use of megafauna for habitat definition

**Figure 4:**
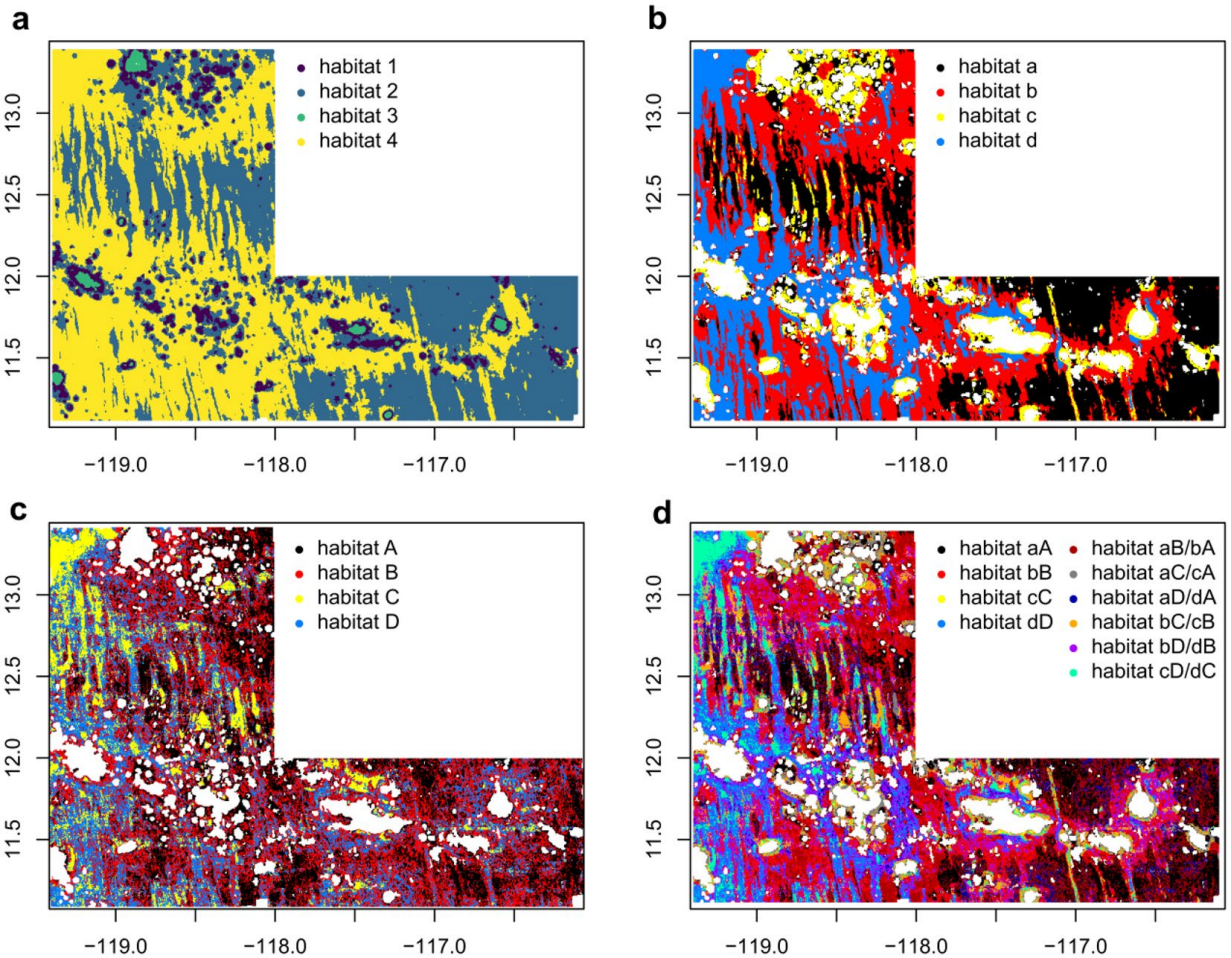
Habitat maps of the German contract area computed with k-means clustering for megafauna morphotypes, bathymetric variables and backscatter value, sediment and nodule parameters (**a**), only including megafauna morphotypes, bathymetric variables and backscatter value and excluding seamount sites (**b**), for meiofauna abundance, bathymetric variables and backscatter value as computed by Uhlenkott et al.^22^ with k-means clustering (**c**) and differences between the habitat maps computed for meiofauna and megafauna excluding seamount sites (**d**). Habitat map for meiofauna obtained from https://doi.org/10.1594/PANGAEA.912217^32^.

Predicted probabilities of occurrence of the megafauna morphotypes were combined with environmental data (e.g. bathymetry, sediment and nodule characteristics) and these variables were clustered with k-means clustering, replicating an approach previously conducted on meiofauna^22^. These clusters are named habitat maps in this approach in the sense of a biotope, connecting information on the benthic fauna with environmental variables. The Calinski criterion^33^ suggested a division of the area into four habitats as an ideal solution. Habitat 3 mainly includes seamount summits, positioned within a seamount area in the north and a seamount chain from west to east (Fig. 4a). Habitat 1 includes the flanks and surroundings of seamounts as well as abyssal hills (Fig. 4a). Habitats 2 and 4 are distributed across the abyssal plains, habitat 2 being prevalent in the south-eastern part of the study area and habitat 4 being most abundant in the western part of the area in the vicinity of seamount areas (Fig. 4a).

Computing a habitat map for megafauna including bathymetry and backscatter values but excluding all sites with a water depth of less than 3000 m (i.e. seamount sites, as these have a clearly different assemblage and may bias habitat mapping), the Calinski criterion^33^ suggested a division into two clusters at most. However, to directly compare the habitat map produced here to a previously published habitat map for meiofauna (Fig. 4c)^32^, four clusters were used to enable a point-to-point comparison (Fig. 4b). In this exercise, habitat c is mainly distributed in the direct vicinity of seamount sites. The seamount-based habitat c is surrounded by habitat b in the north-east of the study area, and by habitat d at the south-western seamount chain (Fig. 4b). Habitat a is prevalent in plain areas, mainly positioned in the south-east of the study area (Fig. 4b). Compared to the habitat map for meiofauna (Fig. 4c), the four megafauna habitats are more clearly differentiated (Fig. 4b).

Comparing the habitat maps for megafauna and meiofauna, the area assigned to habitat d in the megafauna-based habitat map (Fig. 4b) is covered by meiofauna habitats C and D (Fig. 4d). Habitat A and habitat B reflect an alternating dominance in the south-eastern part of the area (Fig. 4d). Although seamounts do appear to have an influence on the classification and extent of the meiofauna habitat, no individual habitat was assigned only to seamount sites (Fig. 4c); hence, habitat c in the megafauna-based habitat map is alternating dominance with the distribution of habitat C and B in the meiofauna-based habitat map (Fig. 4d).

## Discussion

The clustering approach reveals clear patterns in megafaunal distribution, structuring the study area into three distinct megafauna assemblages. Mainly, these assemblages comprise an eastern and a western plain area, and an additional area encompassing bathymetric elevations such as seamounts and abyssal hills. In the context of deep-sea nodule mining, the benthic community is susceptible to potentially severe impacts related to the mining process^10^; however, the three distinct assemblages will be affected by mining activities differently. As mining will typically be conducted in the abyssal plains with low slope angles, the two assemblages described from the abyssal plains are representative for potential mining sites. For these, the delineation of protected areas as well as preservation reference zones is of utmost importance for conservation as well as for monitoring^1,18,21,22^. The distinctly different assemblage described from seamounts and abyssal hills will not be directly endangered by the mining process, but indirect influences such as the spreading sediment plume^34^ or metal mobilization^35^ have the potential to also affect these areas. Therefore, a potential preservation reference zone not only needs to contain identical assemblages or habitats compared to the directly impacted area, but also counterparts for areas that may be affected indirectly.

When the APEIs in the CCZ were defined, the inclusion of seamounts into these protected areas was strongly recommended^1^, firstly because seamounts are often described as hotspots of biodiversity^15,36^, but also because they often provide hard substratum^37^. In our study, the predicted richness (derived from the predicted probability of morphotype occurrences) is highest in areas of bathymetric elevation (i.e. seamounts and abyssal hills; assemblage 1 in Fig. 1c). A potential reason is the enhanced habitat heterogeneity due to differences in flux of particulate organic carbon (POC) and the current regime at seamounts^37^, but differences in depth, slope and particle grain size distribution may also cause significant differences in megafaunal density and biomass even at abyssal hills compared to plain sites (e.g. in the north-eastern Atlantic^38^). However, in accordance with Cuvelier et al.^17^, our study shows that seamounts in the German contract area do harbour a distinctly different community compared to nodule field areas nearby (both hills and plains) and, hence, they do not appear to be a suitable refuge for the megabenthic biodiversity found in deeper, nodule-bearing areas, as has been observed in other areas of the CCZ^39,40^. Still, it has to be considered that all images used in this study were obtained at abyssal hills and the lower flanks of seamounts and therefore an additional megafaunal assemblage might occur at seamount summits.

Lowest megafauna richness is predicted in areas surrounding the seamount sites and being prevalent in the western part of the study area (assemblage 3 in Fig. 1c). These areas have slightly greater water depths compared to their surroundings, including ring-like depressions surrounding the seamounts that have formed as a result of isostatic adjustment as well as N-S trending troughs (and hills) in the western part of the study area. In a study comparing the megafaunal community of a trough area observed in APEI-6 (north of our study area) to nearby flat and ridge sites, megafaunal density was lower in the trough area^5^. However, the actual richness directly observed at positions representative of assemblage 3 was higher compared to the total richness of the images obtained within assemblage 1, although this is likely to be biased by the higher number of images assigned to assemblage 3. Assemblage 2, also distributed across the abyssal plains but at a slightly shallower water depth compared to assemblage 3, exhibited a higher number of observed specimens at a similar sampling size.

There are several possible explanations for the relatively high abundance and richness of assemblage 2 in the eastern part of the study area compared to the western part. Firstly, the CCZ exhibits a decrease in particulate organic carbon (POC) flux from east to west^41,42^. Most areas in the deep sea solely rely on the input of organic material from the sea-surface as food source^43,44^, which sinks in the form of POC through the water column^45^. Low food availability is known to be a factor that reduces megafaunal density^46^, and could explain at least some of the W–E differences observed in the area. Another explanation might be that the water depth of these two plain assemblages is close to the carbonate compensation depth (CCD) in the study area^12^, with the western assemblage being slightly deeper. On one site, the non-availability of calcium carbonate might exclude the occurrence of some morphotypes that either directly (e.g. shells) or indirectly (e.g. food) depend on its availability. The CCD also influences the sediment composition due to the dissolution of carbonate tests and has also been hypothesized to influence formation and size of the polymetallic nodules^12^, which are known to influence density and diversity of the benthic megafauna of the CCZ^8,13^.

There is a striking correlation between the modelled megafauna distribution / morphotype richness of assemblage 2 (Fig. 1a), with highest richness in the eastern abyssal plains, and habitat 3 in Fig. 4a, which in addition to megafauna takes bathymetric variables and backscatter value, sediment and nodule parameters into account. This habitat 3 primarily consists of contiguous nodule fields with only small or gradual changes in elevation and reflects the seafloor areas that are most prospective for potential future mining^47^. In the western plains that are crossed by N–S trending hills and ridges, nodule fields are interspaced with outcrops and nodule-free sediments and are thus much less contiguous. The lower richness in trough and depression areas might be a result of the typically lower nodule presence in these seabeds, potentially also due to coverage by sediment slides, which might suppress the amount and type of nodule substrate that is available for megafauna.

Compared to habitat maps based on meiofauna abundance^22^, megafaunal assemblages show clearer differences in distribution across the study area. Potentially, the more patchy distribution of meiofauna can be traced back to the very high variability of meiofauna abundance on a small scale, whilst showing low variability in composition at a high taxonomic level and at a regional scale^22,30^. Abundance of megafauna is distinctly lower than that of meiofauna, with usually less than 1 animal per square metre in the CCZ (e.g.^4^). Therefore, megafauna is more directly influenced by large-scale patterns such as differences in bathymetry^5^. Furthermore, modelling of meiofauna was based on abundances, whereas in this study the modelling approach was performed as a classification on presence-absence data. Using this type of data inhibits the investigation of dominant morphotypes and does not allow the delineation of areas with high megafauna abundance. Another difference between the habitat maps based on meio- and megafauna is the taxonomic scale, i.e. the higher taxonomic resolution in megafauna data, that enables a sharper detection of niches for megafauna morphotypes compared to higher meiofauna taxa. The higher meiofauna taxa can be observed in most deep-sea sediments in all world oceans. Some megafauna species like the the ophiuroid *Ophiosphalma glabrum* (Lütken & Mortensen, 1899) (corresponding to morphotype code OPH_010 in this study) can also have a large distribution range^48^, but the observation of megafauna species can also be limited e.g. indicating potential endemism at seamounts^36^.

These differences in distribution patterns are also likely to influence model performance of the individual morphotypes (s. Supplementary Table S1). Hence, the error is small for morphotypes such as the morphotype *Hymenaster* sp. indet. mtp-AST_017, which occurs in high abundances and is strongly bound to the assemblage covering seamount and hill areas, but distinctly higher for taxa occurring in lower abundances and in a less clear pattern. In this context, Valvani et al. stated that not only class imbalance, i.e. few observations within a dataset contrasting a large proportion of background data where a taxon is absent, but also class overlap can impair performance of random forest^49^. Additionally, distribution of sampling efforts adds an additional level of spatial imbalance as only few samples were obtained in the western plain areas compared to the east. Hence, only general trends can be obtained both from the predicted distribution as well as the derived assemblage and habitat maps. Further sampling, especially in the west of the study area, might significantly increase model performance and prediction.

All in all, the clear division of the benthic megafaunal community across the study area facilitates the use of distribution models and clustering methods to define potential protected areas or preservation reference zones based on a benthic community that is as similar as possible to the community of a potentially impacted area. Moreover, the results of this study show that neither seamounts and their surroundings, nor troughs, hills or depressions will suffice as reference zones for prospective nodule mining areas, as morphotype richness is generally lower in those areas. Furthermore, the differences between the habitat maps based on megafauna and meiofauna show that multiple characteristics of the benthic community should be compared to cover as many properties of the benthic ecosystem as possible to effectively implement conservation approaches.

## Material and methods

**Figure 5:**
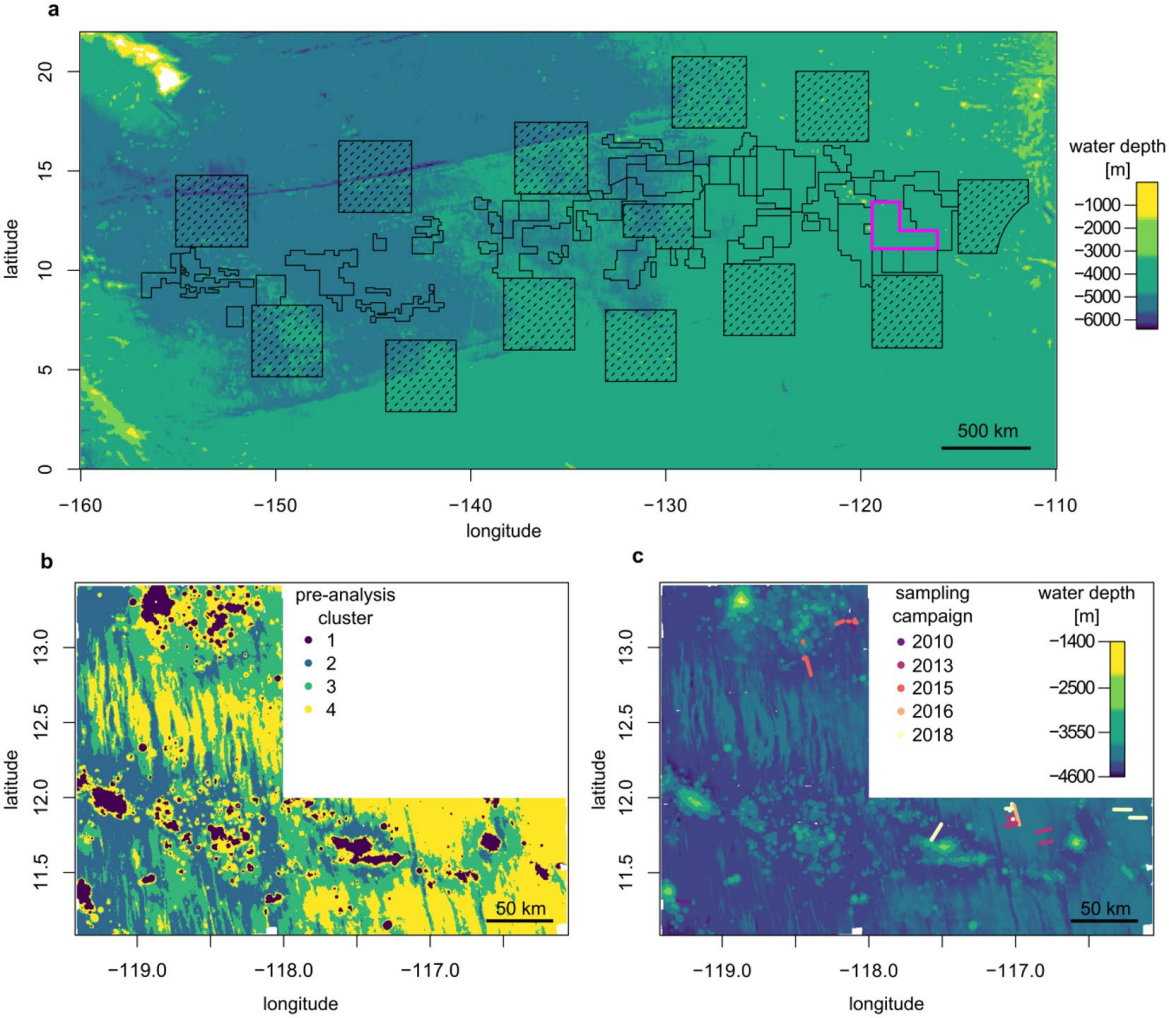
Bathymetric map of the Clarion Clipperton Fracture Zone in the north-east Pacific with the study area, which is the German contract area for polymetallic nodule exploration, marked in magenta (**a**); a more detailed bathymetric map of the German contract area including the positions of the deep-sea images used in this study obtained at five cruises between 2010 and 2018 (**c**); distribution of the k-means clusters based on bathymetry used to obtain random subsamples of images including different bathymetric conditions (**b**).

The study area is the German contract area for the exploration of polymetallic nodules in the eastern CCZ, with a size of approximately 62,000 km^2^ (Fig. 5). Seabed imagery obtained using different towed-camera platforms during five cruises between 2010 and 2018 and along 196 km of seafloor photographic transects (s. Supplementary Table S1) was used to investigate the invertebrate metazoan megabenthic community. For analysis, only geo-referenced images for which the covered seabed area could be scaled were used. Overlapping images were removed based on image position and size of the covered seabed to avoid double counting of fauna.

To avoid bias resulting from different sampling intensities in the study area, the area was divided into four different clusters based on bathymetry (Fig. 5b). Therefore, continuous layers of water depth and backscatter value with a resolution of 715 m^50^ were clustered together with the bathymetric derivatives slope, aspect and bathymetric position index (BPI) at two scales (1 km and 17 km) (s. Supplementary Table S5). All bathymetric derivatives were computed using the R-package *raster*^51^. Clustering was conducted using the CLARA algorithm (clustering for large applications)^52^ as implemented in the R-package *cluster*^53^, manually choosing four clusters as an intermediate amount of division. To have an appropriate number of images representing the bathymetric conditions of the study area, images were subset randomly for each bathymetric cluster based on the percentage of spatial coverage of the respective cluster across the study area (Table 2). Only 303 images were available for cluster 1 representing 6% of the study area; the number of images for the other clusters was adapted accordingly (Table 2).

**Table 2:** Area coverage and number of images used from the division of the study area based on clustering of bathymetric parameters.

Cluster	1	2	3	4
Area coverage	6%	24%	28%	36%
Number of images in study	303	1212	1414	1818

Detection and taxonomic classification of megafauna individuals from seabed images was conducted in the BIIGLE annotation system^54^ as set up on the Server of the Senckenberg Nature Research Society (SGN). Invertebrate metazoan megafauna specimens were identified to the lowest taxonomic hierarchy possible (morphotype; typically family or genus level) in accordance with an abyssal-Pacific standardized taxa catalogue derived from previous image-based studies conducted in the region (see e.g.^4,5,55^) and by reference to existing literature (e.g.^56–58^). The taxonomic nomenclature of the morphotypes was adapted as suggested by Horton et al.^59^. Invertebrates living in a shell or tube (e.g. most polychaete and gastropod taxa) were excluded from the analyses as in most cases it is not possible to determine whether these are alive based on image data. Similarly, megafaunal-sized xenophyophore tests were not included in the analyses as it is not possible to determine if these are alive from the images alone^60^.

For the computation of distribution models, occurrences per image were converted to presence-absence data. The presence-absence matrix was used to compute random forest classification^23^ as implemented in the R package *randomForest*^61^ with default settings for each morphotype that occurred at more than 10 images within the dataset individually. Bathymetry and backscatter value were used as predictor variables, as well as environmental variables of the sediment and the nodules spatially predicted as a grid based on their values in boxcore samples (s. Supplementary Table S5); all data were used with a resolution of 121 m. The random forest models were used to predict the probability of the occurrence of each morphotype spatially across the study area. From the predicted distribution maps, assemblages and habitat maps were computed with k-means clustering using the function *cascadeKM* from the R package *vegan*^62^. The ideal number of clusters was determined based on the Calinski criterion^33^, which regards the ratio of the dispersion between clusters to the dispersion within clusters. For the comparison of the resulting megafauna habitat map to a previously published habitat map of meiofauna^32^, more clusters were used than suggested by the Calinski criterion, which suggested the lowest number of clusters possible, to enable the direct comparison of the predicted habitats of both faunal size classes. For the same reason, spatial resolution was reduced to 715 m in the predictions.

All computations were conducted in the statistical environment of R^63^ using, in addition to the packages *cluster*, *randomForest*, *raster* and *vegan*, the R packages *reshape2*^64^, *plyr*^65^, *viridisLite*^66^, *rangeBuilder*^67^ and *Ternary*^68^.

## Supplementary Information

Supplementary Tables.

## Data Availability

The megafauna annotations as well as the spatially predicted probability of occurrences will be stored in the PANGAEA data publisher and information system.
