# Supplementary material for: Investigating the benthic megafauna in the eastern Clarion Clipperton Fracture Zone (north-east Pacific) based on distribution models predicted with random forest
Source: Sci Rep. 2022 May 17;12:8229. doi: 10.1038/s41598-022-12323-0 (PMC9114404; doi:10.1038/s41598-022-12323-0)
Supplement: Supplementary file 1 — Supplementary Tables. [file 41598_2022_12323_MOESM1_ESM.docx]

**Supplementary information:**

Table S1: Out of bag (OOB) error for the random forest classifications based on presence-absence data of 68 megafauna morphotypes occurring at least at 10 locations; additionally the number of occurrences is given for the three assemblages derived from the predicted probabilities of occurrences across the study area.

| taxonomic nomenclature | morphotype code | OOB general | OOB absence | OOB presence | occurrences per assemblage | | |
| --- | --- | --- | --- | --- | --- | --- | --- |
|  |  |  |  |  | 1 | 2 | 3 |
| Actiniaria mtp-ACT_002 | ACT_002 | 0.31 | 0.31 | 0.63 | 0 | 13 | 3 |
| Actiniaria mtp-ACT_004 | ACT_004 | 0.21 | 0.21 | 0.5 | 0 | 31 | 8 |
| Actiniaria mtp-ACT_010 | ACT_010 | 0.34 | 0.34 | 0.53 | 0 | 11 | 4 |
| Actiniaria mtp-ACT_016 | ACT_016 | 0.23 | 0.23 | 0.7 | 0 | 13 | 8 |
| Actiniaria mtp-ACT_022 | ACT_022 | 0.19 | 0.18 | 0.56 | 4 | 39 | 34 |
| Actiniaria mtp-ACT_023 | ACT_023 | 0.28 | 0.27 | 0.87 | 3 | 7 | 5 |
| Actiniaria mtp-ACT_061 | ACT_061 | 0.19 | 0.19 | 0.83 | 3 | 3 | 6 |
| Actiniaria mtp-ACT_068 | ACT_068 | 0.24 | 0.24 | 0.54 | 0 | 9 | 4 |
| Primnoidae mtp-ALC_002 | ALC_002 | 0.28 | 0.28 | 0.3 | 4 | 0 | 6 |
| *Bathygorgia* sp. mtp-ALC_003 | ALC_003 | 0.24 | 0.23 | 0.48 | 7 | 16 | 4 |
| *Bathygorgia profunda* sp. inc. | ALC_004 | 0.23 | 0.23 | 0.42 | 9 | 84 | 26 |
| *Lepidisis* sp. indet. mtp-ALC_005 | ALC_005 | 0.18 | 0.18 | 0.57 | 23 | 20 | 15 |
| *Calyptrophora persephone* sp. inc. | ALC_007 | 0.24 | 0.24 | 0.39 | 13 | 17 | 4 |
| *Abyssoprimnoa* *gemina* sp. inc. | ALC_008 | 0.28 | 0.28 | 0.34 | 67 | 173 | 131 |
| *Callozostron* *bayeri* sp. inc. | ALC_009 | 0.25 | 0.24 | 0.5 | 37 | 126 | 96 |
| Taiaroidae mtp-ALC_030 | ALC_030 | 0.21 | 0.2 | 0.75 | 3 | 15 | 10 |
| Isididae mtp-ALC_035 | ALC_035 | 0.23 | 0.23 | 0.6 | 3 | 6 | 1 |
| Primnoidae mtp-ALC_038 | ALC_038 | 0.24 | 0.23 | 0.49 | 67 | 73 | 54 |
| Primnoidae mtp-ALC_039 | ALC_039 | 0.14 | 0.14 | 0.37 | 26 | 10 | 7 |
| Polynoidae mtp-ANN_003 | ANN_003 | 0.2 | 0.2 | 0.27 | 5 | 3 | 3 |
| *Abyssopathes* *lyra* sp. inc. | ANT_002 | 0.27 | 0.26 | 0.69 | 7 | 62 | 70 |
| *Bathypathes* sp. indet. mtp-ANT_003 | ANT_003 | 0.24 | 0.23 | 0.87 | 1 | 14 | 8 |
| Antipatharia mtp-ANT_006 | ANT_006 | 0.26 | 0.26 | 0.7 | 1 | 10 | 10 |
| Scalpellidae mtp-ART_010 | ART_010 | 0.26 | 0.26 | 0.71 | 2 | 8 | 4 |
| *Freyastera* sp. indet. mtp-AST_002 | AST_002 | 0.23 | 0.22 | 0.69 | 14 | 20 | 17 |
| Paxillosida mtp-AST_004 | AST_004 | 0.25 | 0.24 | 0.39 | 18 | 89 | 194 |
| *Hyphalaster* sp. indet. mtp-AST_007 | AST_007 | 0.26 | 0.26 | 0.87 | 0 | 6 | 9 |
| *Hymenaster* sp. indet. mtp-AST_017 | AST_017 | 0.05 | 0.05 | 0.04 | 238 | 3 | 1 |
| Asteroidea mtp-AST_037 | AST_031 | 0.24 | 0.24 | 0.64 | 3 | 13 | 9 |
| *Smithsonius* sp. indet. mtp-BRY_001 | BRY_001 | 0.2 | 0.19 | 0.43 | 26 | 42 | 17 |
| *Notoplites* sp. indet. mtp-BRY_002 | BRY_002 | 0.17 | 0.17 | 0.18 | 7 | 36 | 12 |
| *Columnella* sp. indet. mtp-BRY_003 | BRY_003 | 0.26 | 0.26 | 0.36 | 6 | 44 | 7 |
| Bryozoa mtp-BRY_007 | BRY_007 | 0.11 | 0.11 | 0.36 | 12 | 2 | 1 |
| Bryozoa mtp-BRY_009 | BRY_009 | 0.2 | 0.2 | 0.29 | 1 | 16 | 0 |
| Bryozoa mtp-BRY_012 | BRY_012 | 0.2 | 0.2 | 0.29 | 22 | 28 | 2 |
| Spirularia mtp-CER_001 | CER_001 | 0.21 | 0.21 | 0.53 | 9 | 7 | 4 |
| Ceriantharia mtp-CER_008 | CER_008 | 0.13 | 0.13 | 0.29 | 0 | 23 | 3 |
| *Corallimorphus* sp. indet. mtp-COR_001 | COR_001 | 0.29 | 0.29 | 0.62 | 9 | 29 | 15 |
| Corallimorpharia mtp-COR_003 | COR_003 | 0.19 | 0.19 | 0.63 | 4 | 8 | 4 |
| *Glyphocrangon* sp. indet. mtp-DEC_006 | DEC_006 | 0.29 | 0.29 | 0.82 | 1 | 10 | 6 |
| *Hymenopenaeus nereus* sp. inc. | DEC_008 | 0.23 | 0.23 | 0.3 | 3 | 0 | 7 |
| *Hyalonema* sp. indet. mtp-HEX_002 | HEX_002 | 0.19 | 0.19 | 0.7 | 3 | 19 | 11 |
| *Bathydorus laniger* sp. inc. | HEX_007 | 0.29 | 0.29 | 0.65 | 1 | 11 | 5 |
| *Sympagella clippertonae* sp. inc. | HEX_008 | 0.25 | 0.25 | 0.64 | 2 | 15 | 5 |
| *Docosaccus maculatus* sp. inc. | HEX_015 | 0.21 | 0.2 | 0.77 | 5 | 36 | 23 |
| *Holascus euonyx* sp. inc. | HEX_019 | 0.21 | 0.21 | 0.67 | 5 | 7 | 6 |
| *Bathydorus laniger* sp. inc. | HEX_033 | 0.25 | 0.24 | 0.6 | 0 | 11 | 5 |
| *Mesothuria* sp. indet. mtp-HOL_001 | HOL_001 | 0.21 | 0.21 | 0.65 | 2 | 9 | 15 |
| *Synallactes* sp. indet. mtp-HOL_007 | HOL_007 | 0.24 | 0.24 | 0.66 | 0 | 30 | 34 |
| *Synallactes* sp. indet. mtp-HOL_008 | HOL_008 | 0.23 | 0.22 | 0.53 | 5 | 17 | 10 |
| *Benthodytes incerta* sp. inc. | HOL_041 | 0.24 | 0.24 | 0.21 | 10 | 44 | 5 |
| *Synallactes* sp. indet. mtp-HOL_104 | HOL_104 | 0.22 | 0.22 | 0.74 | 0 | 11 | 8 |
| *Ophiacantha* mtp-OPH_001 | OPH_001 | 0.34 | 0.34 | 0.59 | 0 | 12 | 7 |
| Ophiacanthidae mtp-OPH_002 | OPH_002 | 0.27 | 0.27 | 0.41 | 0 | 19 | 11 |
| Ophiopyrgidae mtp-OPH_003 | OPH_003 | 0.06 | 0.06 | 0.07 | 284 | 5 | 2 |
| *Ophiosphalma* *glabrum* sp. inc. | OPH_010 | 0.35 | 0.3 | 0.5 | 72 | 917 | 617 |
| Ophiopyrgidae mtp-OPH_014 | OPH_014 | 0.3 | 0.3 | 1 | 0 | 9 | 5 |
| Ophiuroidea mtp-OPH_015 | OPH_015 | 0.2 | 0.19 | 0.6 | 11 | 19 | 22 |
| Ophiuroidea mtp-OPH_020 | OPH_020 | 0.25 | 0.24 | 0.43 | 1 | 8 | 5 |
| Ophiuroidea mtp-OPH_021 | OPH_021 | 0.27 | 0.27 | 0.68 | 5 | 11 | 9 |
| Ophiuroidea mtp-OPH_022 | OPH_022 | 0.28 | 0.27 | 0.55 | 11 | 82 | 39 |
| Demospongiae mtp-POR_002 | POR_002 | 0.28 | 0.27 | 0.52 | 15 | 100 | 137 |
| *Hyalonema (Onconema) clarioni* sp. inc. | POR_022 | 0.32 | 0.32 | 0.67 | 1 | 8 | 3 |
| *Hyalonema (Corynonema) depressum* sp. inc. | POR_033 | 0.35 | 0.35 | 0.59 | 4 | 17 | 6 |
| *Hyalonema (Prionema) breviradix* sp. inc. | POR_046 | 0.31 | 0.31 | 0.64 | 0 | 11 | 4 |
| Porifera mtp-POR_101 | POR_101 | 0.2 | 0.2 | 0.35 | 0 | 14 | 6 |
| *Pleisiodiadema globosum* sp. inc. | URC_003 | 0.22 | 0.21 | 0.38 | 10 | 124 | 176 |
| Echinoidea mtp-URC_012 | URC_012 | 0.14 | 0.13 | 0.59 | 9 | 6 | 8 |

Table S2: Pairwise permutational analysis of variance (PERMANOVA) (https://github.com/pmartinezarbizu/pairwiseAdonis) comparing the three megafauna assemblages (Figure 1b); p-value adjusted according to the Bonferroni correction.

| pair | F-model | R^2^ | p-value | adj. p-value |
| --- | --- | --- | --- | --- |
| 3 vs 2 | 140.62618 | 0.050976890 | 0.001 | 0.003* |
| 3 vs 1 | 124.72382 | 0.045557487 | 0.001 | 0.003* |
| 2 vs 1 | 22.11411 | 0.005167917 | 0.001 | 0.003* |

Table S 3: Metadata of the photographic surveys conducted during the five cruises.

| cruise | research vessel | year | towed camera system | abbreviation | distance laser pointers | image resolution (px x px) | mean altitude (or range) | mean covered area | citation |
| --- | --- | --- | --- | --- | --- | --- | --- | --- | --- |
| SO-205 | RV Sonne | 2010 | Ocean Floor Observation System | OFOS | 20 cm | 4224 x 2376 | ~2 m | 3.65±1.08 | Rühlemann, C. & Shipboard Scientific Party. Cruise report SO205 MANGAN, Hannover, Germany. (2010). |
| KM-13 | RV Kilo Moana | 2013 | Multi-Functional Tool | MFT | 50 cm | 4000 x 3000 | ~3-6 m | 2.08±0.59 | Rühlemann, C. & Shipboard Scientific Party. Cruise Report MANGAN 2013, Hannover, Germany. (2014). |
| SO-240 | RV Sonne | 2015 | Simpler TauchROboter Modular ERweiterbar | STROMER | 30 cm | 4000 x 3000 | ~1.5 -4 m | 2.33±0.84 | Kuhn, T. & Shipboard Scientific Party. Cruise report SO240 FLUM, Hannover, Germany. (2015). |
| KM-16 | RV Kilo Moana | 2016 |  |  |  | 4000 x 3000 | ~3-6 m | 4.53±1.94 | Rühlemann, C. & Shipboard Scientific Party. Cruise report MANGAN 2016, Hannover, Germany. (2017). |
| SO-268 | RV Sonne | 2018 |  |  |  | 4000 x 3000 | ~3 m | 5.39±1.64 | Rühlemann, C. & Shipboard Scientific Party. Cruise report MANGAN 2018, Hannover, Germany. (2019). |

Table S 4: Numbers of images selected for the pre-analysis cluster based on bathymetric variables and backscatter value, and properties of the variables used for clustering within each cluster.

| cluster | total images | images in subset | water depth [m] | backscatter value | slope [radians] | aspect [radians] | BPI (1 km) | BPI (17 km) |
| --- | --- | --- | --- | --- | --- | --- | --- | --- |
| 1 | 323 | 323 | 1536-4006 | 137.0±16.5 | 0.19±0.11 | 3.05±1.80 | 240.8±213.0 | 133.6±210.0 |
| 2 | 4.280 | 2.023 | 4322-4556 | 97.9±16.3 | 0.03±0.04 | 3.19±1.74 | -53.3±51.4 | -32.2±60.6 |
| 3 | 5.383 | 2.336 | 4229-4325 | 99.7±16.6 | 0.04±0.04 | 3.15±1.75 | -22.7±47.1 | -13.6±64.9 |
| 4 | 19.565 | 2.572 | 3997-4236 | 105.8±19.5 | 0.06±0.07 | 3.09±1.76 | 17.4±63.7 | 12.6±96.5 |

Table S 5: Predictor variables used for the random forest classification of the 68 most abundant megafauna morphotypes and for the computation of habitat maps with k-means clustering.

| environmental variable | source | resolution | used for random forest classification | used for k-means clustering |
| --- | --- | --- | --- | --- |
| water depth | Wiediecke-Hombach & shipboard scientific party^53^ | 121 m/715 m | x | x |
| backscatter | Wiediecke-Hombach & shipboard scientific party^53^ | 121 m/715 m | x | x |
| slope | *terrain()* | 121 m/715 m | x | x |
| eastness | *terrain()* | 121 m/715 m | x | x |
| northness | *terrain()* | 121 m/715 m | x | x |
| flow direction | *terrain()* | 121 m/715 m | x | x |
| bathymetric position index (BPI) 300 m | *focal()* | 121 m/715 m | x | x |
| bathymetric position index (BPI) 1.5 km | *focal()* | 121 m/715 m | x | x |
| bathymetric position index (BPI) 5 km | *focal()* | 121 m/715 m | x | x |
| roughness | *terrain()* | 121 m/715 m | x | x |
| terrain ruggedness index (TRI) | *terrain()* | 121 m/715 m | x | x |
| dry bulk density (1 cm) | Uhlenkott et al.^25^ | 121 m | x |  |
| dry bulk density (4 cm) | Uhlenkott et al.^25^ | 121 m | x |  |
| shear strength (2 cm) | Uhlenkott et al.^25^ | 121 m | x |  |
| silt | boxcore sediment/ *randomforest()* | 121 m | x |  |
| total carbon (1 cm) | Uhlenkott et al.^25^ | 121 m | x |  |
| total carbon (4 cm) | Uhlenkott et al.^25^ | 121 m | x |  |
| total inorganic carbon (1 cm) | Uhlenkott et al.^25^ | 121 m | x |  |
| total inorganic carbon (4 cm) | Uhlenkott et al.^25^ | 121 m | x |  |
| total organic carbon (1 cm) | Uhlenkott et al.^25^ | 121 m | x |  |
| weight nodules | boxcore nodules/ *randomforest()* | 121 m | x |  |
| wet weight nodules | Uhlenkott et al.^25^ | 121 m | x |  |
| number nodules | Uhlenkott et al.^25^ | 121 m | x |  |
| mean size nodules | Uhlenkott et al.^25^ | 121 m | x |  |
| ratio large (>4 cm) to small (<4 cm) nodules | Uhlenkott et al.^25^ | 121 m | x |  |
| content barium | Uhlenkott et al.^25^ | 121 m | x |  |
| content copper | Uhlenkott et al.^25^ | 121 m | x |  |
| content iron | Uhlenkott et al.^25^ | 121 m | x |  |
| content lithium | Uhlenkott et al.^25^ | 121 m | x |  |
| content molybdenum | Uhlenkott et al.^25^ | 121 m | x |  |
| content nickel | Uhlenkott et al.^25^ | 121 m | x |  |
| content titanium | Uhlenkott et al.^25^ | 121 m | x |  |
| content zinc | Uhlenkott et al.^25^ | 121 m | x |  |
| content zirconium | Uhlenkott et al.^25^ | 121 m | x |  |
| quotient of manganese and iron | Uhlenkott et al.^25^ | 121 m | x |  |
| sum of rare earth elements | Uhlenkott et al.^25^ | 121 m | x |  |
